# Physical activity and trajectories of frailty among older adults: Evidence from the English Longitudinal Study of Ageing

**DOI:** 10.1371/journal.pone.0170878

**Published:** 2017-02-02

**Authors:** Nina T. Rogers, Alan Marshall, Chrissy H. Roberts, Panayotes Demakakos, Andrew Steptoe, Shaun Scholes

**Affiliations:** 1 Department of Epidemiology & Public Health, University College London, London, United Kingdom; 2 Department of Geography and Sustainable Development, Irvine Building, University of St Andrews, St Andrews, Fife, Scotland, United Kingdom; 3 Clinical Research Department, Faculty of Infectious and Tropical Diseases, London School of Hygiene & Tropical Medicine, London, United Kingdom; Nathan S Kline Institute, UNITED STATES

## Abstract

**Background:**

Frail older adults are heavy users of health and social care. In order to reduce the costs associated with frailty in older age groups, safe and cost-effective strategies are required that will reduce the incidence and severity of frailty.

**Objective:**

We investigated whether self-reported intensity of physical activity (sedentary, mild, moderate or vigorous) performed at least once a week can significantly reduce trajectories of frailty in older adults who are classified as non-frail at baseline (Rockwood’s Frailty Index [FI] ≤ 0.25).

**Methods:**

Multi-level growth curve modelling was used to assess trajectories of frailty in 8649 non-frail adults aged 50 and over and according to baseline self-reported intensity of physical activity. Frailty was measured in five-year age cohorts based on age at baseline (50–54; 55–59; 60–64; 65–69; 70–74; 75–79; 80+) on up to 6 occasions, providing an average of 10 years of follow-up. All models were adjusted for baseline sex, education, wealth, cohabitation, smoking, and alcohol consumption.

**Results:**

Compared with the sedentary reference group, mild physical activity was insufficient to significantly slow the progression of frailty, moderate physical activity reduced the progression of frailty in some age groups (particularly ages 65 and above) and vigorous activity significantly reduced the trajectory of frailty progression in all older adults.

**Conclusion:**

Healthy non-frail older adults require higher intensities of physical activity for continued improvement in frailty trajectories.

## Introduction

Frailty is a common and yet complex geriatric condition that is characterised by failure of multiple physiological systems together with reduced capacity to resist minor stressors [1]. Frail adults are at increased risk of adverse health outcomes including cognitive decline, falls, disability, institutionalisation and hospitalisation [1–3]. While ageing and frailty are intrinsically linked, frailty is superior to age when used to predict health outcomes including both sustained wellbeing [4] and survival [5]. A recent study examining the impact of frailty on hospital admission in England between 2005 and 2013 revealed that increasing numbers of over 65 year olds were being admitted to hospital, with falls and cognitive impairment being the most prevalent conditions reported [6]. Population ageing and the consequential challenge to health and social care in older adults has prompted a growing interest in frailty and how it can be ameliorated through cost-effective and accessible interventions. The benefits to health of maintaining a physically active lifestyle are well accepted and there is good evidence that physical activity (PA) can delay the onset [7,8] and dampen the progression [7,9] of frailty in older adults. The association between PA and frailty differs widely between studies because there is no single definition of frailty [10] and it is common for studies to use different indicators to define frailty. PA-based interventions have also yielded conflicting results in older frail adults and there is uncertainty surrounding the most advantageous intensity, frequency and duration of PA for adults of different ages and with differing degrees of frailty [10–12]. In the United Kingdom (UK), current PA guidelines for healthy adults aged 65 and over suggest strength exercises at least twice a week plus either 150 minutes of moderately intense PA or 75 minutes of vigorous PA, or a combination of the two [13].

In this study we have utilised data from a well-established panel study, the English Longitudinal Study of Ageing (ELSA), to investigate whether different intensities of PA performed at least once a week attenuate the progression of frailty over a period of 10 years in older adults.

## Materials and methods

Sample: The data used in this study were collected from ELSA, a nationally representative panel study of men and woman aged 50 and over in 2002 and living in private households in England [14]. Participants in ELSA were initially recruited from households that had participated in the Health Survey for England (HSE) in 1998, 1999 and 2001. Participants were followed up every two years allowing for repeated measures to be recorded in the same individuals over time. ELSA participants provided written informed consent, and the London Multi-Centre Research Ethics Committee granted ethical approval.

### Measures

#### Exposure

Participants were asked at wave 1 (2002–2003) to indicate how often they participated in vigorous, moderate, and mild physical activities during their leisure time, for which the options were: (a) more than once per week, (b) once per week, (c) one to three times per month, or (d) hardly ever. To assist participants in deciding the level of intensity of their own leisure activities, they were shown prompt cards with examples of activities and their associated intensities. Examples of mild PA included vacuuming, home repairs and laundry. Examples of moderate PA included washing the car, dancing, floor/stretching exercises, walking at a moderate pace and gardening. Vigorous PA examples included running or jogging, cycling, tennis, swimming and digging with a spade. These questions on physical activity status were extracted from a validated physical activity interview and they have been used previously in the HSE physical activity assessment [15]. The highest intensity of PA that was carried out at least once per week was used to define how active a participant was. PA status at baseline was categorized into four mutually exclusive groups: sedentary; mild; moderate; and vigorous [16].

#### Outcome

The frailty index (FI) was created using the procedures of Rockwood and colleagues [17]. The FI included 56 variables that covered a range of domains, conditions and systems including disability (activities of daily living and instrumental activities of daily living), mobility, cognitive function, self-rated health, vision, hearing and chronic diseases including cardiovascular diseases and depression. Guidelines for utilising variables to generate a score on the FI were taken from the literature [17] and were as follows: (a) variables must be associated with health and generally increase with age; (b) the variables must not be too common and saturate the adult population too early; (c) they must cover a range of systems and processes in the body; and (d) since the same people are examined over time, the same variables that are included in the FI at baseline must be used in all waves of the study [17]. The majority (51/56) of variables were dichotomised so that a score of 1 was assigned for every deficit that was present and a score of 0 given for every deficit that was absent. Deficits that were not fully expressed were assigned an intermediate score of between 0 and 1. An FI of between 0 and 1 was created for each individual by totaling the number of deficits they reported and dividing that by the total number of deficits that were being evaluated.

The FI is unstable if the number of potential deficits considered is too small, however the inclusion of at least 30 deficits has been reported as sufficient to accurately predict adverse outcomes [17]. Importantly, frailty is defined more precisely by the number of deficits accumulated rather than their precise nature and it has been shown that within a population, different sample deficits can be selected at random with no significant effect on the predictive power of the FI provided that the number of deficits being considered are sufficient [9]. Data from individuals with missing information on any of the 56 variables could therefore still be used to generate the FI as long as they had complete data for at least 30/56 variables [18]. At baseline an FI with a score of 0.25 or less was used to define an individual as non-frail; this cut-point has previously been used in the literature [19].

#### Covariates

Well-described factors affecting health were included as baseline covariates in the analysis. These included sex, educational qualifications (no qualifications, O-levels or A-levels, degree/higher or equivalent), total non-pension wealth (quintiles), being married or cohabiting, current smoking status (a smoker/non-smoker) and alcohol intake (drinking almost daily).

### Statistical analysis

Sample characteristics (age, sex, wealth quintiles, highest formal educational qualification, living alone, alcohol intake, current smoking status and baseline PA) were estimated using percentages for variables with categorical data; means and standard deviations were used for variables with continuous data. The ELSA data comprises of repeat observations within the same individuals; multilevel modelling is particularly suitable for examining frailty trajectories in this sample since it allows for the issue of non-independence of an individual’s score on the FI over time. A further advantage of the model is that it can handle unequal intervals between observations as well as missing data.

The aim of the analysis was to determine whether cohort-specific trajectories of frailty, over a period of 10 years, differ according to baseline intensity of PA. We used multilevel growth curve models to estimate changes in FI scores as a function of time (i.e. survey wave entered as a continuous variable) in seven five-year age cohorts (50–54; 55–59; 60–64; 65–69; 70–74; 75–79; 80+) in the four baseline PA groups.

**Fig 1:**
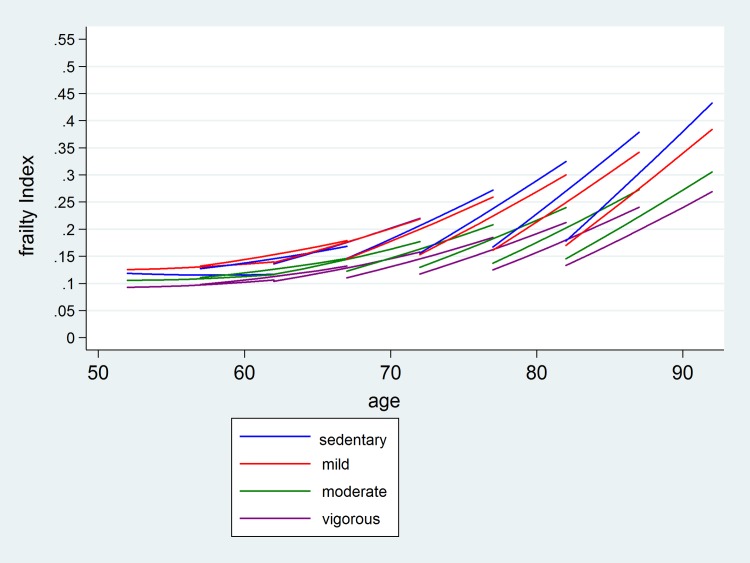
Average eleven-year frailty trajectories in five-year age cohorts (50–54; 55–59; 60–64; 65–69; 70–74; 75–79; 80+) of non-frail adults, predicted by baseline physical activity status. FI = frailty index.

Baseline PA was entered as a categorical variable (sedentary group as reference). The full model included main effects for survey wave, age cohort and baseline PA, two-way interaction terms (wave × age cohort; wave × PA; age cohort × PA), and three-way interactions (age cohort × wave × PA). The three-way interaction terms allow the age cohort-specific trajectory of frailty to vary according to the four baseline PA groups (or alternatively, to allow the PA-specific trajectory of frailty to vary according to the 7 age-cohorts). A quadratic term for time and age cohort was included in the model to allow for non-linear trajectories of frailty and non-linear growth in frailty across age-cohorts respectively. Survey waves 1–6 were zero-centred on wave three so that time was entered into the models using the values -2, -1, 0, 1, 2 and 3. Analysis was repeated by using uncentered waves, using the values 0, 1, 2, 3, 4, 5 corresponding to waves 1, 2, 3, 4, 5 and 6, respectively. Estimates from the full model were used to graphically show the frailty trajectories for each combination of baseline PA and cohort (Fig 1).

To ease interpretation of the full model, we ran 7 models with age-cohort-specific analyses that included the two-way interaction terms between survey wave and PA. A statistically significant interaction term indicates a difference in the rate of progression between a specific PA group and the reference PA group (i.e. sedentary). A negative coefficient indicates that, compared to the reference group, we observe a slower progression of frailty within a particular PA category (e.g. the group participating in vigorous activities) with increasing time, with a positive coefficient showing the opposite. All analyses were performed using Stata14 (StataCorp LP, College Station, Texas).

## Results

### Data

The original sample at baseline consisted of 11,391 participants. There were 10,524 individuals who had non-missing data for baseline level physical activity status, all covariates and a sufficient number of variables (at least 30 out of a possible 56 variables) to generate a score on the frailty index [17]. Of the 10,524 individuals, 8,649 (82%) were classified as non-frail at baseline (FI ≤ 0.25) and were included in the analytical sample. As expected with longitudinal data, there were reductions in the size of the sample over time; of the 8,649 participants in the analytical sample at wave 1, 6991 (81%), 6086 (70%), 5465 (63%), 5178 (60%) and 4,776 (55%) took part at waves 2, 3, 4, 5 and 6, respectively. The number of waves that (n) participants took part in were as follows; one wave: 1,207 (14.0%); two waves: 974 (11.3%); three waves: 840 (9.7%); four waves: 746 (8.7%); five waves: 806 (8.6%) and six waves: 4,076 (47.1%). Out of a possible six waves, individuals participated in an average of 4.3 waves. Baseline characteristics of the study sample are presented in Table 1. Non-frail participants had an average FI score of 0.11; or 6 deficits out of the total of 56 deficits considered. The average age of the sample was 64 years old. Slightly more females (53%) than males were present, and 27.9% of the sample lived alone and 37.7% of the sample possessed no formal educational qualifications. In terms of health behaviours, 30% of the sample consumed alcohol daily, and 13.9% reported being current smokers. A minority of the sample reported being sedentary (5.3%) or partaking in mild intensity (11.4%) physical activities at least once a week. The majority of older adults reported that they took part in moderate (51.5%) or vigorous (31.8%) physical activity at least once a week.

**Table 1:** Baseline sample characteristics (N = 8,649).

	Non-Frail (FI <0.25) N = 8649	
Frailty Index–mean (SD)	0.11	(0.06)
Age–mean (SD)	64.0	(9.74)
Female- N (%)	4597	(53.2)
Poorest wealth quintile–N (%)	1728	(20.0)
No educational qualifications-N (%)	3261	(37.7)
Living alone–N (%)	2410	(27.9)
Drinking alcohol almost daily—N %	2592	(30.0)
Smoker- N %	1206	(13.9)
Baseline physical activity		
Sedentary -N (%)	462	(5.3)
Mild -N (%)	986	(11.4)
Moderate -N (%)	4451	(51.5)
Vigorous -N (%)	2750	(31.8)

The prevalence of the different variables that were used to generate the FI is shown in Table 2. The mobility components, which included difficulties climbing several flights of stairs and stooping, kneeling and crouching were common (>25%) amongst non-frail adults at baseline. Other commonly reported conditions and difficulties included high blood pressure (34.6%), restless sleep (34.3%), feeling sad (15.0%) much of the time, and poor/fair as opposed to good/ very good/ excellent self-reported general health (16.4%).

**Table 2:** Prevalence of baseline FI components, in non-frail adults.

Domain	Frailty components	Non-Frail (N = 8649)	
		N	%
Mobility Difficulties	Walking 100 yards	241	2.79
	Sitting for about two hours	643	7.44
	Getting up from a chair after sitting for long periods	1396	16.1
	Climbing several flights of stairs without resting	2177	25.2
	Climbing one flight of stairs without resting	407	4.71
	Stooping, kneeling, or crouching	2179	25.2
	Reaching or extending arms above shoulder level	414	4.79
	Pulling or pushing large objects like a living room chair	591	6.83
	Lifting or carrying weights over 10 pounds, like a heavy bag	1211	14.0
	Picking up a 5p coin from a table	156	1.79
Disability (ADL/iADL)	Dressing, including putting on shoes and socks	431	5.00
	Walking across a room	19	0.22
	Bathing or showering	281	3.25
	Eating, such as cutting up your food	25	0.29
	Getting in or out of bed	99	1.15
	Using the toilet, including getting up or down	47	0.54
	Using a map to figure out how to get around in a strange place	209	2.42
	Preparing a hot meal	36	0.42
	Shopping for groceries	123	1.42
	Making telephone calls	54	0.62
	Taking medication	26	0.30
	Managing money, (e.g. paying bills and keeping track of expenses)	48	0.56
	Doing work around the house or garden	447	5.17
General health	Self-reported general health (fair/poor compared to excellent/very good/ good)	1417	16.4
Depressive Symptoms	Respondent felt depressed much of the time during past week	986	11.5
	Respondent felt that everything they did during the past week was an effort	1194	14.0
	Respondent felt that their sleep was restless during the past week	2934	34.3
	Respondent was not happy much of the time during the past week	617	7.23
	Respondent felt lonely much of the time during the past week	791	9.24
	Respondent did not enjoy life much of the time during the past week	494	5.79
	Respondent felt sad much of the time during the past week	1288	15.0
	Respondent could not get going much of the time during the past week	1134	13.3
Self-reported conditions	High blood pressure or hypertension	2994	34.6
	Angina	564	6.52
	Heart attack (including myocardial infarction or coronary thrombosis)	356	4.12
	Congestive heart failure	31	0.36
	An abnormal heart rhythm	461	5.33
	Diabetes or high blood sugar	489	5.67
	A stroke (cerebral vascular disease)	196	2.27
	Chronic lung disease such as chronic bronchitis or emphysema	352	4.07
	Asthma	847	9.80
	Arthritis (including osteoarthritis, or rheumatism)	2209	25.6
	Osteoporosis, sometimes called thin or brittle bones	265	3.07
	Cancer or a malignant tumour (excluding minor skin cancers)	479	5.54
	Parkinson's disease	20	0.23
	Any emotional, nervous or psychiatric problems	508	5.88
	Alzheimer's disease	2	0.02
	Dementia, organic brain syndrome, senility or other serious memory impairment	12	0.14
	Eyesight (while using lenses, if appropriate) poor compared to excellent	153	1.77
	Hearing (while using hearing aid, if appropriate) poor compared to excellent	298	3.45
Cognitive function	Successfully identified today's date	1471	17.1
	Successfully identified today's month	163	1.90
	Successfully identified today's year	155	1.81
	Successfully identified the day of the week	137	1.60
	Immediate word recall (lowest quintile)	1916	22.5
	Delayed word recall (lowest quintile)	2140	27.2

To ease interpretation of the full model, the coefficient terms in Table 3 represent the cohort-specific analyses highlighting the difference in the rate of change in the frailty score over 1-wave between participants in each PA group (mild; moderate; vigorous) compared to participants in the reference PA group (sedentary). A negative interaction term indicates a slower progression in frailty for a particular PA group compared with the reference category (sedentary). Compared to adults who were sedentary at baseline, adults who reported mild PA at least once a week at baseline did not show any improvement in the course of frailty at any age (Table 3).

**Table 3:** Trajectories of frailty in participants (FI < = 0.25 at baseline), predicted by physical activity at baseline.

Physical activity status for change in frailty (non-frail at baseline)										
	Vigorous			Moderate			Mild			Sedentary
Age group	β Coefficient (95% CI)	P	N	β Coefficient (95% CI)	P	N	β Coefficient (95% CI)	P	N	N
50–54	-0.031 (-0.046, -0.016)	<0.0001	671	-0.021 (-0.036, -0.007)	0.004	805	-0.006 (-0.017, 0.016)	0.95	151	55
55–59	-0.025 (-0.040, -0.011)	0.001	666	-0.009 (-0.024, 0.005)	0.21	900	0.009 (-0.007, 0.026)	0.30	146	61
60–64	-0.019 (-0.035, -0.004)	0.012	448	-0.007 (-0.021, 0.008)	0.38	694	0.013 (-0.004, 0.030)	0.12	129	60
65–69	-0.040 (-0.054, -0.027)	<0.0001	445	-0.034 (-0.047, -0.021)	<0.0001	703	-0.008 (-0.024, 0.008)	0.31	125	72
70–74	-0.036 (-0.053, -0.019)	<0.0001	274	-0.028 (-0.044, -0.012)	0.001	592	0.001 (-0.017, 0.018)	0.95	147	62
75–70	-0.044 (-0.062, -0.025)	<0.0001	163	-0.024 (-0.042, -0.007)	0.005	411	-0.005 (-0.015, 0.023)	0.64	125	58
80+	-0.061 (-0.081, -0.042)	<0.0001	83	-0.039 (-0.054, -0.023)	<0.0001	346	-0.014 (-0.031, 0.004)	0.13	163	94

Estimates were obtained from mixed models including survey wave, baseline PA, and their interaction. The table shows the coefficients for the survey wave by baseline PA interaction term. The interaction term shows the estimated difference in the 1-wave rate of change between participants in the relevant PA group and participants in the sedentary group.

Moderate PA at least once a week was associated with improved frailty progression of the cohorts aged 65 and over as well as those aged 50–54. A greater improvement in frailty progression occurred in adults who reported vigorous PA at least once a week. Analysis was repeated using an un-centred time variable, which yielded estimates that were almost identical to when time was centred (available on request). Fig 1 depicts vector graphs of the model frailty trajectories in 5-year age cohorts predicted by baseline physical activity status.

The estimated coefficients that were used to specify the frailty trajectories are shown in S1 Table. Interestingly, the frailty gap between recent and earlier age-cohorts appears to be largest in those participants that were classified as sedentary at baseline and smallest in those reporting moderate or vigorous PA at least once a week

## Discussion

The findings from this study offer evidence that PA may be an effective way of dampening the course of frailty in older English adults. The intensity of PA that is performed at least weekly is shown to be a vital factor in the relationship between PA and frailty.

For non-frail adults who are age 50 or over, mild PA at baseline is insufficient to improve frailty progression compared with being sedentary. The propensity of PA to significantly improve frailty progression in older adults who are non-frail appears to be confined to more intensive PA. Vigorous PA that is carried out at least once a week appears to be the most effective means to reduce the progression of frailty in older ages. These results are in line with previous studies that have reported a reduction in the incidence and progression of disabilities [20] and improvement in physical functioning [11] when higher intensities of PA are performed. In light of these findings on frailty trajectories in older adults, current UK PA guidelines appear to be appropriate: however further emphasis on the importance of regular vigorous PA should be considered.

Mild PA, compared to sedentary behaviour, was not associated with improving frailty trajectories in non-frail adults, perhaps because these non-frail adults experience ceiling effects on the magnitude of frailty improvement. By their very nature, non-frail adults have accrued a limited number of health deficits over their life course. To an extent the expression of some deficits is likely to be organic and an inevitable part of the normal ageing process, and is not reversible or significantly improved by increasing weekly PA status from a sedentary state to mild PA. Most but not all non-frail age groups appear to benefit from moderate PA compared with being sedentary. Frailty trajectories do however appear to be improved in all adults aged 65 and over and this might be explained by considering that older adults above 65 and over are likely to have accrued higher levels of frailty and therefore have more potential for improvement than adults aged under 65.

It has been demonstrated that different variables carry varying degrees of importance in explaining differences in frailty amongst older persons; physical strength, energy and mobility have been shown to contribute the most in explaining differences in frailty status and it is possible that these features of frailty may be particularly responsive to PA [21,22]. We show that some mobility issues are commonly experienced by participants and this has previously been reported in those who are frail [23]. Further studies are necessary to determine whether the components of frailty are all of equal importance in terms of responsiveness to PA.

Consistent with other studies [24,25], we show that more recently born cohorts of older adults are frailer compared with earlier born cohorts. In a previous study [24], the authors found that wealth differences were responsible for much of the increase in frailty levels in more recent born cohorts and it was suggested that reductions in the level of PA, particularly across the poorest age cohorts might be one driver. The graphical results of our analysis suggest that PA is indeed a driver of the frailty differences between different age-cohorts that is documented in the literature. Further investigation into the lifestyles and changing characteristics of the population will be necessary to further explore these cohort differences.

The inevitable increase in frailty over the adult lifespan could however mean that adults that are approaching older ages need different considerations such as what types of PA are most suitable, because they are likely to suffer a wider range of issues ranging from strength, cognitive and sensory impairment. It has been demonstrated however that regular physical activity is safe for both frail and non-frail [26] adults and studies using resistance training [27] and physical therapy [28] have shown that even adults who are deemed frail are not at significantly higher risk of adverse events. Challenges do however exist in encouraging frailer adults to become and remain active, for example there is a strong relationship between degree of hindrance caused by a condition, its prognosis and compliance in an exercise regime [29]. Performance and adherence for example, have also been shown to be worse if too many exercises are added to the regime [30]. More research is needed to determine the optimum level of PA required plus the best frequency and duration of PA in adults who are deemed to be frail.

Strengths of this study include the large size of the study sample and high follow-up rates, which enhance the generalizability of the study results. The availability of data for up to 6 observations per participant over an average period of 10 years presents an informative picture of how scores on a well-developed frailty index change over time and how this change over time varies by intensity of weekly PA.

While our findings demonstrate that the trajectories of frailty appear to be influenced by baseline PA we cannot prove causality. It may be that the respondents who take part in more intensive PA are healthier to begin with in some unobserved way at baseline and it is this underlying health advantage, rather than the level of PA, that results in their less steep frailty trajectories. The findings from this study are however limited to adults who are non-frail at baseline and to some extent this guards against the possibility of reverse causality; that those who are physically active are able to undertake activity because they have better underlying health and so experience a slower increase in frailty as a result. However, we cannot rule out the possibility of a causal link in the opposite direction to that hypothesised. Examination of PA intensity over multiple time-points (rather than at baseline alone) would be useful in the assessment of whether a sustained rather than a short-lived period of physical activity is of additional benefit in terms of frailty progression in older adults. A recent study which also used the ELSA data showed that PA levels in older English adults were fairly stable across the various waves [31]. In light of this finding, we have operationalised the baseline PA data as a fair approximation of long term PA behaviours, without compromising the protection against problems relating to reverse causality that come from using multiple waves of PA data.

One way to extend this analysis would be to examine adults who were frail at baseline and the benefits of PA for such groups. Such analysis is important but challenging because very few adults who were frail participated in more vigorous activities and this was particularly true for the older age groups.

The assessment of PA was based on self-reported measures and older adults tend to over-estimate their true levels of PA [32]. It is therefore possible that our findings underestimate the true relationship between the intensity of weekly physical activity and frailty. Our study did not capture information on interventions that might lead to improvements in health status and which could lead to an over-estimation of the effect of the intensity of weekly PA on frailty trajectories. As the length of follow-up increases, the loss of participants who are less healthy means that the average health of the population moves in a favourable direction. Thus our findings are likely to be generalizable to healthy older adults, and our estimates might reflect conservative estimates of the range of increase in frailty index scores over the 10-year period.

Future studies that feature exercise type, duration, frequency and intensity are needed to build on our understanding of how PA impacts on frailty and a consensus on which criteria should be used to define frailty is also necessary, not least because a larger number of studies could then be combined and utilised in meta-analysis to produce studies that are powered to analyse adults of different ages and with different degrees of frailty.

## Conclusion

Our study emphasises that PA interventions and guidelines for older adults should take into account that higher intensities of PA are needed to significantly reduce frailty trajectories in the individuals and communities that are targeted. Vigorous and moderate PA should be encouraged throughout later life and particularly at the oldest ages.

### Disclaimer

The funders had no role in the study design; in the collection, analysis and interpretation of data; in writing of the report; or in the decision to submit the paper for publication. The developers and funders of ELSA and the Archive do not bear any responsibility for the analyses or interpretations presented here.

## Supporting information

S1 Table Growth curve models of frailty and ageing by physical activity intensity carried out at least weekly. *P<0.05; **p<0.01; ***p<0.001.
(DOCX)
