# Supplementary material for: Physical activity and trajectories of frailty among older adults: Evidence from the English Longitudinal Study of Ageing
Source: PLoS One. 2017 Feb 2;12(2):e0170878. doi: 10.1371/journal.pone.0170878 (PMC5289530; doi:10.1371/journal.pone.0170878)
Supplement: S1 Table — *P<0.05; **p<0.01; ***p<0.001. (DOCX) [file pone.0170878.s001.docx]

**S1 Table:** Growth curve models of frailty and ageing by physical activity intensity carried out at least weekly.

*P<0.05; **p<0.01; ***p<0.001

|  | **Cohort trajectories** |
| --- | --- |
| **Intercept** | **0.19 ***** |
| **Cohort** | **0.027***** |
| **Cohort^2** | **0.0003** |
| **Growth rate** | **0.025 ***** |
| **Growth rate^2** | **0.0004 ***** |
| **Growth rate * cohort** | **0.009 ***** |
|  |  |
| **Mild PA** | **-0.005 ***** |
| **Moderate PA** | **-0.039 ***** |
| **Vigorous PA** | **-0.056 ***** |
|  |  |
| **Mild PA * cohort** | **-0.006**** |
| **Moderate PA * cohort** | **-0.010***** |
| **Vigorous PA * cohort** | **-0.012***** |
|  |  |
| **Mild PA * Growth rate** | **-0.002** |
| **Moderate PA * Growth rate** | **-0.008 ***** |
| **Vigorous PA * Growth rate** | **-0.010 ***** |
|  |  |
| **Mild PA * cohort * Growth rate** | **-0.002*** |
| **Moderate PA * cohort * Growth rate** | **-0.004***** |
| **Vigorous PA * cohort * Growth rate** | **-0.004***** |
| **Random effects** |  |
| **Level 1: within person** | **0.002***** |
| **Level 2: In intercept** | **0.002***** |
| **Level 3: ln growth rate** | **0.0003***** |
